# Construction of 0D/2D Schottky Heterojunctions of ZnO and Ti_3_C_2_ Nanosheets with the Enriched Transfer of Interfacial Charges for Photocatalytic Hydrogen Evolution

**DOI:** 10.3390/ma15134557

**Published:** 2022-06-28

**Authors:** Muhammad Irfan, Irshad Ahmad, Shazia Shukrullah, Humaira Hussain, Muhammad Atif, Stanislaw Legutko, Jana Petru, Michal Hatala, Muhammad Yasin Naz, Saifur Rahman

**Affiliations:** 1 Electrical Engineering Department, College of Engineering, Najran University Saudi Arabia, Najran 61441, Saudi Arabia; miditta@nu.edu.sa (M.I.); srrahman@nu.edu.sa (S.R.); 2 Department of Physics, University of Agriculture Faisalabad, Faisalabad 38040, Pakistan; irshadmahar563@yahoo.com; 3 Department of Chemistry, University of Okara, Punjab 56300, Pakistan; humaira0949@gmail.com; 4 Institute of Chemical Sciences, Bahauddin Zakariya University, Multan 60800, Pakistan; researchismyhobby@gmail.com; 5 Faculty of Mechanical Engineering, Poznan University of Technology, 3 Piotrowo Street, 60-965 Poznan, Poland; stanislaw.legutko@put.poznan.pl; 6 Department of Machining, Assembly and Engineering Metrology, Mechanical Engineering Faculty, VŠB-Technical University of Ostrava, 17. listopadu 2172/15, 708 00 Ostrava, Czech Republic; jana.petru@vsb.cz; 7 Faculty of Production Technologies with a Seat in Prešov, Technical University of Kosice, 1 Bayerova Street, 080 01 Prešov, Slovakia; michal.hatala@tuke.sk

**Keywords:** ZnO, Ti_3_C_2_, 0D/2D heterojunction, hydrogen evolution, photocatalytic activity

## Abstract

The development of cost-effective co-catalysts of high photocatalytic activity and recyclability is still a challenge in the energy transformation domain. In this study, 0D/2D Schottky heterojunctions, consisting of 0D ZnO and 2D Ti_3_C_2_, were successfully synthesized by the electrostatic self-assembling of ZnO nanoparticles on Ti_3_C_2_ nanosheets. In constructing these heterojunctions, Ti_3_C_2_ nanosheets acted as a co-catalyst for enhancing the transfer of excitons and their separation to support the photocatalytic response of ZnO. The as-prepared ZnO/Ti_3_C_2_ composites demonstrate an abbreviated charge transit channel, a huge interfacial contact area and the interfacial electrons’ transport potential. The extended optical response and large reactive area of the ZnO/Ti_3_C_2_ composite promoted the formation of excitons and reactive sites on the photocatalyst’s surface. The ZnO/Ti_3_C_2_ Schottky heterojunction showed significantly high photocatalytic activity for hydrogen production from a water–ethanol solution under the light illumination in the visible region. The hydrogen evolution overoptimized the ZnO/Ti_3_C_2_ composition with 30 wt.% of Ti_3_C_2_, which was eight times higher than the pristine ZnO. These findings can be helpful in developing 0D/2D heterojunction systems for photocatalytic applications by utilizing Ti_3_C_2_ as a low-cost co-catalyst.

## 1. Introduction

The ever-worsening energy problem caused by the rapid depletion of non-renewable fossil fuels has prompted researchers to develop photocatalysts that convert unlimited sunlight straight into H_2_ fuel via photocatalytic water splitting [1,2]. Usually, the process of photocatalytic H_2_ evolution involves three processes, namely, (i) the formation of excitons after absorbing photons of energy exceeding the band gap energy of the catalyst, (ii) the separation and transportation of photoinduced e^−^/h^+^ pairs towards the surface of the catalyst; and (iii) the contribution of charge carriers in the redox reaction at surface-active sites to produce H_2_ [2]. The H_2_ fuel, being pollution free, sustainable and renewable, has earned a widespread focus as a substitute for conventional fossil fuels due to its maximum energy gradient among all the available chemical fuels. The efficiency of the hydrogen evolution reaction via water splitting greatly depends on the optical response range, recombination rate of excitons and redox capacity of the considered photocatalyst [3]. The recent decade has experienced massive development in nanomaterials used to explore an efficient and industrial-scale outlet for photocatalytic H_2_ evolution. Up to now, numerous strategies and pathways have been adopted to search for economical, stable and wide light-responsive candidates for H_2_ evolution. Several materials such as titanium oxide (TiO_2_) [4], cerium oxide (CeO_2_) [5], zinc sulfide (ZnS) [6], graphitic carbon nitride [7], zinc oxide (ZnO) [8], cadmium sulfide (CdS) [9], etc., have been extensively explored for their performance and viability in this emerging field. Among semiconductors, ZnO reveals diversified morphologies and an appropriate optical band gap (~3.37 eV) to effectively harvest the sunlight [10]. Although ZnO has shown highly photocatalytic efficiency for different environment treatment and energy applications, the pristine ZnO possesses some unavoidable constraints such as a visible light absorption inability, the swift recombination of excitons, photo-corrosion after light exposure and an insufficient number of active sites, which consequently reduces its utilization for industrial-scale H_2_ evolution [8,9,10,11].

To address the outlined issues, diverse approaches, including elemental doping [12], loading of the co-catalyst [13], integrating with other semiconductors [14], etc., have been dynamically explored. Despite noteworthy advancements during the last decades in the betterment of photocatalytic H_2_ evolution efficiency via the nano-structuring of ZnO [15], the accurate development of a ZnO-modified composite outlet is still a high challenge. Moreover, the present photocatalytic H_2_ generating ZnO-based candidates do not satisfy the targeted expectations owing to the technical barrier in simultaneously enhancing the photocatalytic performance and stability and reducing the high price affiliated with costly noble metal co-catalysts [16]. A fruitful strategy for addressing the issue is to integrate ZnO with a conductive noble metal co-catalyst such as Pt, Au, etc., to design strong and integrated hybrid photocatalytic frameworks with an inhibited recombination of excitons, rapid transmission of charge carriers and the availability of numerous catalytic sites to induce swift redox reactions to trigger the H_2_ evolution process [17]. Despite obtaining much higher photocatalytic H_2_ evolution performance due to the utilization of these noble metal co-catalysts, the overpriced cost and extensive scarcity greatly restrict their large-scale application. Therefore, the search for an inexpensive and noble metal-free co-catalyst is essential in order to promote the activity of a photocatalyst for hydrogen evolution.

Among ultrathin MXenes, titanium carbide (Ti_3_C_2_) has emerged as a hot photocatalytic material owing to its strong conductivity, broad light-harvesting ability, enriched surface hydrophilic groups and strong reactive capacity stemming from the disclosed terminal metal sites [18]. The aforesaid unique features of Ti_3_C_2_ make it highly appropriate for designing high-performance Ti_3_C_2_-modified hybrid photocatalysts. Considering the promising characteristics of 0D and 2D nanomaterials, the synthesis of the 0D ZnO/2D Ti_3_C_2_ composite system for obtaining large-scale photocatalytic activity is appealing and significantly predicted. Li et al. [19] performed a photocatalytic reduction of CO_2_ over Ti_3_C_2_/ZnO composites. The revealed reduction efficiency of the composite photocatalyst was higher than the pristine ZnO due to the swift transfer of electrons towards the co-catalyst Ti_3_C_2_ [19]. Similarly, the ZnO/Ti_3_C_2_ composite has also been documented as improving the photocatalytic degradation of methylene blue [20]. However, the construction of 0D/2D ZnO/Ti_3_C_2_ heterojunction systems for the photocatalytic conversion of water into hydrogen fuel has not been documented well in the published literature.

Herein, we use 2D Ti_3_C_2_ and 0D ZnO materials to design 0D/2D ZnO/Ti_3_C_2_ composites by the electrostatic assembly route to overcome the limitations of ZnO. The electrostatic interaction between 0D ZnO and 2D Ti_3_C_2_ can result in a strong contact. By the integration of 0D ZnO and 2D Ti_3_C_2_, it is not only the electron-hole recombination rate that can be reduced but also the light absorption and charge transport capacities can be greatly improved compared with those in pure ZnO, resulting in superior photocatalytic H_2_ evolution results.

## 2. Experimental Section

### 2.1. Reagents

Titanium aluminum carbide (Ti_3_AlC_2_, 99.0%) and hydrofluoric acid (HF, 99.9%) were obtained from Sigma-Aldrich, Saint Louis, MO, USA. Dimethyl sulfoxide (DMSO) was bought from Merck. ZnO and DI-water were purchased from Sigma-Aldrich. All chemical agents were used as they were received without performing additional purification procedures.

### 2.2. Preparation of Ti_3_C_2_

The HF etching process was used to prepare Ti_3_C_2_ MXene as follows: 1 g of bulk Ti_3_AlC_2_ powder was steadily added into 20 mL of concentrated hydrofluoric acid (HF, 40%) and placed in oil bath under consistent and vigorous stirring at 60 °C for 48 h to etch the Al layer. The obtained residue was refined with filter paper, centrifuged to eliminate any supernatant and preserved bulk product after centrifugation was cleansed with DI-water successively until the neutral pH was reached. The as-obtained powder was heated at 60 °C in a vacuum furnace for 12 h to obtain few-layered Ti_3_C_2_, which was subsequently redispersed in 20 mL of DMSO and placed under sitting overnight with N_2_ protection. Afterwards, the suspension was centrifuged, cleaned several times with ethanol and DI-water wiped out any remaining DMSO. Subsequently, 0.5 g of collected dry powder was once again re-dispersed in 50 mL of DI-water followed by ultrasonication under N_2_ atmosphere preservation. Following ultrasonication for 60 min, the obtained suspension was centrifuged (3500 rpm, 60 min) to get rid of unexfoliated species. Finally, the black powder of ultrathin Ti_3_C_2_ was obtained. This powder was calcined for 4 h at 700 °C.

### 2.3. Preparation of ZnO/Ti_3_C_2_ Composites

ZnO/Ti_3_C_2_ composites (ZnO/TiC) were synthesized using an electrostatic self-assembly route. Firstly, 3 g of ZnO powder was added to 20 mL of DI-water under constant stirring for one hour, followed by sonication for 20 min to produce a uniform mixture, which was designated as solution A. Next, calculated amounts of Ti_3_C_2_ were dissolved in 20 mL of DI-water with subsequent stirring for 30 min and ultrasonication for 40 min; the solution was labeled as solution B. Afterwards, both solutions were statically dissolved in each other, and the as-obtained suspension was stirred at 2500 rpm for 30 min. The prepared residue was centrifuged to wipe out dispersing species and cleansed with DI-water to obtain the powders, which were dried in an oven at 80 °C for 12 h. Four different ZnO/TiC composites with varying mass content of Ti_3_C_2_ (0.1%, 0.2%, 0.3% and 0.4%) were synthesized by following the same preparation method. The as-prepared composites were labeled as ZnO_0.9_/TiC_0.1_, ZnO_0.8_/TiC_0.2_, ZnO_0.3_/TiC_0.3_ and ZnO_0.96_/TiC_0.4_, respectively, for characterization and photocatalytic activity. 

### 2.4. Characterization

The crystalline structures and phases of the as-prepared composites were recorded over XRD Bruker D8 diffractometer using CuK_α_ radiation of wavelength 0.15046 nm with a scan rate of 2° per min in a 2θ range of 5–80° and V: 40 kV, I: 100 mA. The morphological analysis of as-prepared composites was conducted through scanning electron microscopy (Hitachi S4800, Hitachi, Tokyo, Japan). The optical absorption spectra were recorded using UV-Visible diffuse reflectance spectroscopy (UV-Vis DRS, Perkin Elmer Lambda 950, Waltham, MA, USA) in the range of 200–800 nm with reference calibration in accordance with BaSO_4_. The room temperature photoluminescence spectra were produced with a fluorescence spectrophotometer (Hitachi, F-7000, Hitachi, Tokyo, Japan) in the range of 340–460 nm over an exciton wavelength of 320 nm. BET surface area was measured using BJH modal, NOVA 2200e. The electrochemical impedance spectroscopy was performed with a CH1760E analyzer (frequency: 10 Hz to 1 MHz, light source: 300 Xe lamp with wavelength > 400, the intensity of light: 40 mW·cm^−2^, CH Instruments, Inc., Austin, TX, USA) to determine the charge separation capacity of charge carriers in as-prepared samples. The Mott–Schottky analysis was performed to find out the flat band potentials. The tests were conducted using a conventional three-electrode system where photocatalyst was used to make a working electrode, a platinum foil was used as a counter electrode and Ag/AgCl, immersed in saturated KCl, worked as a reference electrode. Linear sweep voltammetry (LSV) tests were also measured in the same configuration using 1 M KOH as electrolyte. The EIS and Mott–Schottky analyses were carried out in 0.1 M Na_2_SO_2_ solution as electrolyte. The FTO glass, immersed in the photocatalyst, was taken as a working electrode, as reported in our previous study [21].

### 2.5. Photocatalytic Activity

The hydrogen evolution experiments were carried out in a Pyrex reaction with a vessel volume of 100 mL and equipped with a water-cooling system to maintain the temperature of the reaction at 15 °C. Typically, 10 mg of the as-prepared photocatalyst was mixed in 50 mL of DI-water (0.2 gL^−1^) under continuous stirring at 7000 rpm to achieve the homogenous mixture and afterward sonicated for 20 min. Then, the solution was added with 20 vol% of ethanol as holes scavenger. Before illumination to trigger each photocatalytic reaction, the reaction system was fully vacuumed with subsequent bubbling of N_2_ gas for half an hour to completely expel the oxygen gas from the solution. Afterwards, the visible light-driven photocatalytic reaction was commenced under the illumination of 300 W Xe lamp fitted with 400 nm UV cut-off filter and placed 12 cm away from the reactor system. The hydrogen production was quantified using a multi-gas analyzer. The stability experiments were conducted using as-prepared ZnO_0.7_/TiC_0.3_ composite and ethanol was added before and after third cycle.

## 3. Results and Discussion

### 3.1. XRD Structural Analysis

**Figure 1:**
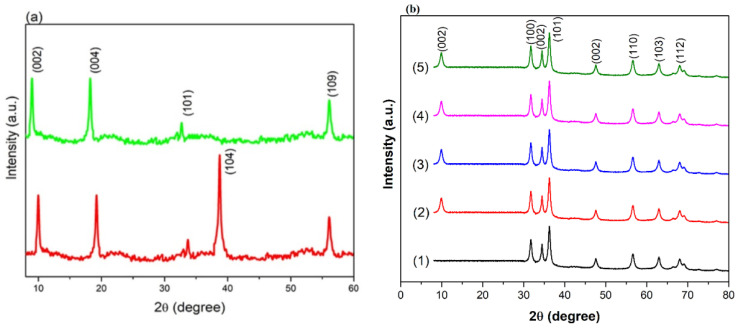
XRD spectra of (**a**) Ti_3_AlC_3_ (red) and Ti_3_C_2_ (green); (**b**) (1) ZnO, (2) ZnO_0.9_/TiC_0.1_, (3) ZnO_0.8_/TiC_0.2_, (4) ZnO_0.7_/TiC_0.3_ and (5) ZnO_0.6_/TiC_0.4_ composites.

Figure 1a displays the XRD patterns of Ti_3_AlC_2_ and Ti_3_C_2_ in the 2θ range of 5–60°. After the HF etching process, the obvious shift of the (002) and (004) diffraction planes from 2θ = 9.54° and 18.8° to 2θ = 8.94° and 17.9°, respectively, and the disappearance of the (104) diffraction planes of Ti_3_AlC_2_ at 2θ = 38.85° authenticated the successful transformation of Ti_3_AlC_2_ to Ti_3_C_2_ [22]. Figure 1b shows that the main diffraction planes (100), (002) and (101) of pristine ZnO are observed at 2θ values of 31.69, 34.44 and 36.34°, respectively, along with other observed peaks at higher 2θ ranges. These planes show the hexagonal wurtzite phase of ZnO, as confirmed from JCPDS 36-1451 [23,24,25]. The XRD profiles of the ZnO/Ti_3_C_2_ composites possess similar XRD patterns to ZnO, with one additional (002) diffraction peak of Ti_3_C_2_. The absence of the remaining diffraction peaks of Ti_3_C_2_ in the XRD patterns of the ZnO/Ti_3_C_2_ composites may be because of a too low intensity of the diffraction peaks of Ti_3_C_2_ compared to pristine ZnO. Moreover, the diffraction peak intensity of the ZnO/Ti_3_C_2_ composites steadily reduced with the increasing content of Ti_3_C_2_ in contrast to the pristine ZnO sample, identifying that the increasing content of Ti_3_C_2_ effectively suppressed the growth of ZnO. These observations strongly confirmed the successful formation of the ZnO/Ti_3_C_2_ composites with varying contents of Ti_3_C_2_.

### 3.2. SEM Analysis

**Figure 2:**
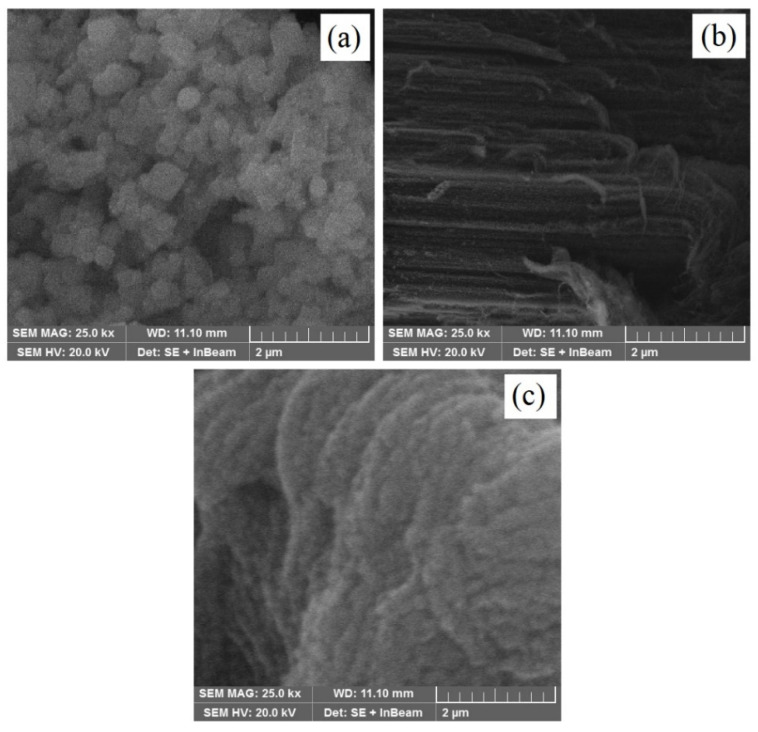
SEM micrographs of (**a**) ZnO nanoparticles, (**b**) Ti_3_C_2_ with conventional 2D-layered structure and (**c**) ZnO/Ti_3_C_2_ composite.

The SEM micrograph of pristine ZnO nanoparticles is shown in Figure 2a, while the SEM micrograph of Ti_3_C_2_ with a conventional 2D-layered structure is shown in Figure 2b. The ultrasonic treatment and calcination destroyed the typical accordion-like morphology of Ti_3_C_2_, which was then modified into different stacked layers with a bed sheets-like morphology, and identified the intuitive fabrication of Ti_3_C_2_, consistent with the XRD results. Moreover, 0D ZnO nanoparticles were observed to be distributed over 2D Ti_3_C_2_, as identified by the SEM micrograph of the ZnO_0.7_/TiC_0.3_ composite, as shown in Figure 2c. The SEM analysis confirmed the integration of the ZnO nanoparticles into Ti_3_C_2_, which provides the rapid separation and transfer of charge carriers. The integration of ZnO and Ti_3_C_2_ results in a high aggregation of ZnO nanoparticles, which, consequently, will provide more active sites.

### 3.3. Optical Absorption

**Figure 3:**
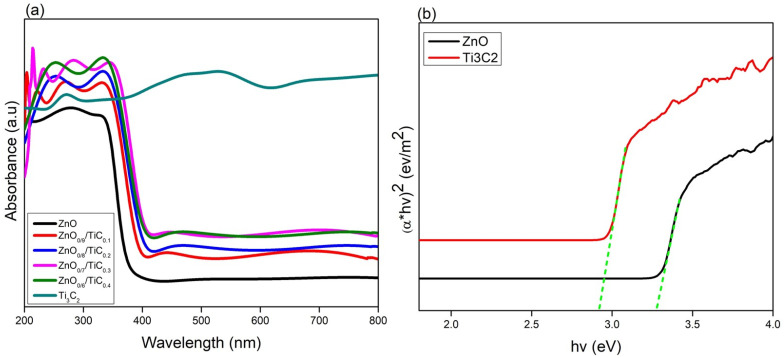
(**a**) UV–Vis spectra of ZnO, Ti_3_C_2_ and ZnO/Ti_3_C_2_ composites; (**b**) Tauc plot of (α*hV)^2^ vs. energy (hV) of ZnO and ZnO_0.7_/TiC_0.3_ composite.

The optical absorption spectra of the as-prepared ZnO, ZnO_0.9_/TiC_0.1_, ZnO_0.8_/TiC_0.2_, ZnO_0.7_/TiC_0.3_ and ZnO_0.6_/TiC_0.4_ composites are displayed in Figure 3a. It is evident that pristine ZnO showed only UV absorption with a cut-off wavelength of 375 nm, consistent with the reported literature [25,26,27,28,29]. With the integration of Ti_3_C_2_, the optical response of ZnO was significantly improved towards the visible region. In contrast to the optical response of pristine ZnO, the ZnO_0.9_/TiC_0.1_ composite revealed high absorption intensity in the UV light spectrum as well as a red shift in the absorption response, identifying that the integration of ZnO and Ti_3_C_2_ significantly improved the photon harvesting capacity, which could be assigned to the black color and metallic nature of Ti_3_C_2_ [29]. The absorption spectra of the ZnO_0.8_/TiC_0.2_ and ZnO_0.7_/TiC_0.3_ composites also demonstrated the steady enhancement in the optical absorption with an obvious red shift towards the visible zone. Moreover, the absorption edge of the ZnO_0.6_/TiC_0.4_ composite showed a blue shift compared to the ZnO_0.7_/TiC_0.3_ composite because of the synergism between ZnO and Ti_3_C_2_ [30].

The Tauc graphs: (α hV)^1/n^ vs. hV (h: Planck constant, V: frequency, α $=\frac{{\left(1-R\right)}^{2}}{2R}$ is the absorption coefficient and n: 1 for the direct band gap (ZnO) and 2 for the indirect band gap (Ti_3_C_2_), respectively) drawn from the UV–Vis absorption data were used for further examination of the as-prepared composites [31]. The linear fit analysis (Figure 3b) showed that all prepared composites possessed the direct band gaps and respective band gap values obtained from the extrapolation of the corresponding tangents of the energy and were found to be 3.31, 3.1, 3.02, 2.92 and 2.98 eV for the bare ZnO, ZnO_0.9_/TiC_0.1_, ZnO_0.8_/TiC_0.2_, ZnO_0.7_/TiC_0.3_ and ZnO_0.6_/TiC_0.4_ composites, respectively.

### 3.4. Spatial Charge Separation and Transfer Ability

**Figure 4:**
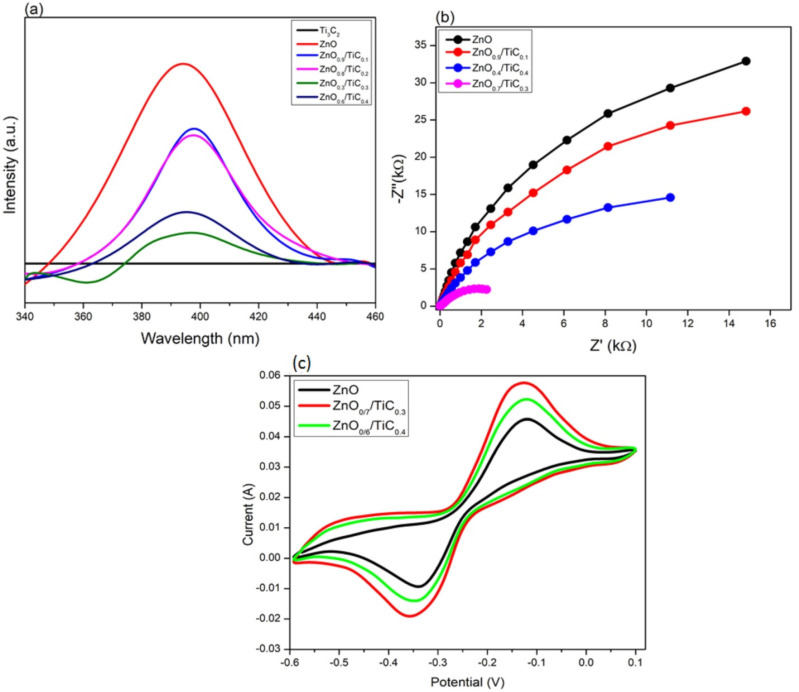
(**a**) PL spectra of ZnO, Ti_3_C_2_ and ZnO/Ti_3_C_2_ composites; (**b**) EIS Nyquist plots of ZnO, ZnO_0.9_/TiC_0.1_, ZnO_0.7_/TiC_0.3_ and ZnO_0.6_/TiC_0.4_; (**c**) CV curves of ZnO, ZnO_0.7_/TiC_0.3_ and ZnO_0.6_/TiC_0.4_.

The transmission and capturing of excitons were further studied by PL fluorescence emission spectra, as reported in Figure 4a. The photocatalyst samples showed a typical ZnO UV emission peak centered around 390 nm due to the fast decay of excitons except in Ti_3_C_2_ [32]. The absence of the emission peak in the PL spectra of Ti_3_C_2_ identified its metallic nature. However, the integration of ZnO with Ti_3_C_2_ greatly lowered the recombination rate of excitons, identifying the effective role of Ti_3_C_2_ to trap the charge carriers. Both the ZnO_0.9_/TiC_0.1_ composite and the ZnO_0.8_/TiC_0.2_ composite exhibited identical PL profiles with red-shifted peaks, identifying the enhanced absorption towards the visible region of the spectrum, in good agreement with the absorption spectra results. Moreover, the ZnO_0.7_/TiC_0.3_ composite demonstrated the lowest emission intensity, which means the lowest recombination of excitons in contrast to other catalysts. Therefore, the PL spectra identified the importance of the construction of heterojunction for suppressing the recombination of excitons and extending the optical response.

To deeply analyze the separation and migration of charge carriers, different characterization such as EIS and CV were also conducted. EIS Nyquist analysis was carried out to further study the separation ad mobility of charge carriers over the as-prepared composites and the results are displayed in Figure 4b. It is evident that the diameters of the Nyquist plots corresponding to the ZnO/Ti_3_C_2_ composites were smaller than bare ZnO, revealing that the integration of Ti_3_C_2_ with ZnO effectively improved the interfacial charge transfer speed due to the high conductivity of Ti_3_C_2_ [33]. Among all the prepared samples, the ZnO_0.7_/TiC_0.3_ composite witnessed the lowest arc radius to identify the highest interfacial separation and mobility of charges, consistent with the PL and UV-Vis results [34]. The CV curves of ZnO, ZnO_0.7_/TiC_0.3_ and ZnO_0.6_/TiC_0.4_ composites were also used to further evaluate the charge migration efficiency, and the results are displayed in Figure 4c. Although all three electrodes exhibited identical redox peaks at the same scan rate and range, the value of the current density was greater for the ZnO/TiC composites in contrast to bare ZnO. The current density for the ZnO_0.7_/TiC_0.3_ composite was obviously higher than ZnO_0.6_/TiC_0.4_, which again confirmed that the most favorable composite with the optimized content of Ti_3_C_2_ was ZnO_0.7_/TiC_0.3_, which can accelerate the transmission of the charge carriers and thereby improve the photocatalytic efficiency towards H_2_ evolution [35].

### 3.5. Photocatalytic H_2_ Evolution Activity and Stability Test

**Figure 5:**
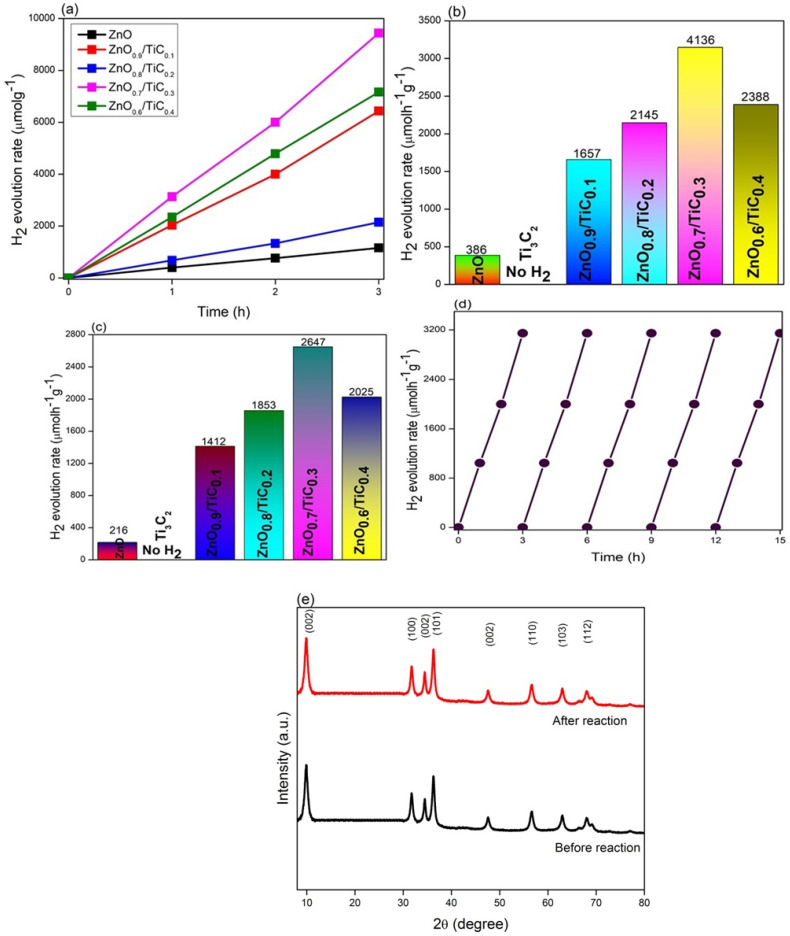
The photocatalytic H_2_ evolution activity of the ZnO and ZnO/Ti_3_C_2_ composites using (**a**,**b**) water–ethanol mixture; (**c**) pure water; (**d**) the photocatalytic H_2_ evolution stability tests of ZnO_0.7_/TiC_0.3_ composite; and (**e**) XRD patterns of ZnO_0.7_/TiC_0.3_ composite before and after reaction.

The photocatalytic capacity of the as-prepared catalysts was evaluated for H_2_ evolution under visible light illumination using ethanol as the sacrificial agent. Figure 5a clearly shows that except for bare Ti_3_C_2_, all other catalysts showed H_2_ evolution from the water–ethanol mixture. For comparison, we also investigated the photocatalytic H_2_ evolution activity of P25 and it showed a photocatalytic result of 0.01 µmol/h/g, which is quite small when compared to other photocatalysts. The bare ZnO catalyst showed a negligible H_2_ evolution rate of 0.03 µmol/h/g, which identified that bare ZnO is inactivated under visible light illumination. In contrast, the construction of the ZnO/TiC composites effectively enhanced the photocatalytic H_2_ evolution even when the introduction of Ti_3_C_2_ was not high. The most optimum composite, ZnO_0.7_/TiC_0.3_ with 30 wt% of Ti_3_C_2_, exhibited more than an eight-fold larger photocatalytic performance than bare ZnO (3146 vs. 386 µmol/h/g). However, further enhancing the content of Ti_3_C_2_ is unfavorable to the photocatalytic H_2_ evolution activity of the ZnO/TiC composite, which reduces to 2388 µmol/h/g in the case of the ZnO_0.6_/TiC_0.4_ composite. The introduction of the suitable content of a metallic nature, Ti_3_C_2_, can promote the performance of charge transfer and separation; however, the number of available catalytic sites and the absorption capacity of ZnO reduces due to the excessive loading of Ti_3_C_2_, which consequently decelerates the photocatalytic activity [36,37].

The photocatalytic H_2_ evolution activity of all the as-prepared catalysts was also evaluated in the absence of a sacrificial agent and the results are displayed in Figure 5b. The H_2_ evolution performance of the catalysts first enhanced and then reduced with the enhancing quantity of T_3_C_2_, which was in line with the H_2_ evolution results from the water–ethanol mixture. Moreover, the H_2_ evolution results of all the prepared samples were much lower than using ethanol as the holes scavenger, which identified the increased productivity of the H_2_ evolution results from the significant role of the sacrificial reagent.

The stability of the as-prepared optimum ZnO_0/7_/TiC_0/3_ catalyst was evaluated for cyclic H_2_ evolution. The H_2_ evolution remained almost unchanged, showing no observable loss in the yield even after 18 h (Figure 5d), which authenticated the fact that the ZnO_0/7_/TiC_0/3_ catalyst was appropriate for photocatalytic H_2_ evolution, and this opens up a new pathway to design and improve the performance of sunlight-driven photocatalysts. Figure 5e shows XRD patterns of the ZnO_0/7_/TiC_0/3_ catalyst composite before and after the photocatalytic H_2_ evolution reaction. It is evident that the XRD patterns did not show any obvious difference, which confirms the strong stability of the ZnO_0/7_/TiC_0/3_ catalyst.

### 3.6. The Mechanism of Improved H_2_ Evolution Activity

**Figure 6:**
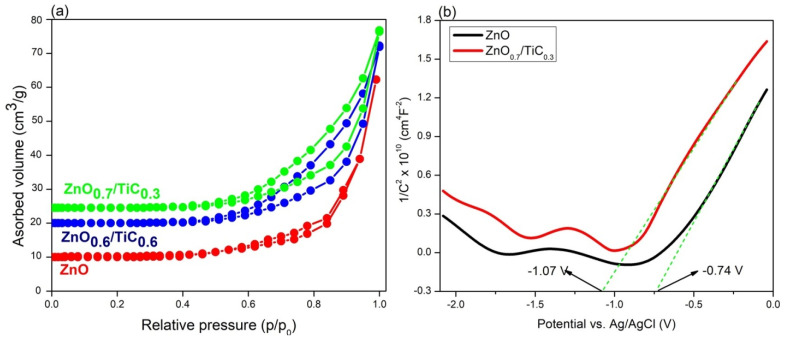
(**a**) Adsorption–desorption isotherms of ZnO, ZnO_0/7_/TiC_0/3_ and ZnO_0/6_/TiC_0/4_ composites; (**b**) Mott–Schottky plots of ZnO and ZnO_0/7_/TiC_0/3_ composites.

The abundance of active sites predominantly controls the rate of the surface redox reactions; therefore, a high surface area of the catalyst is very beneficial for the photocatalytic process [38]. As displayed in Figure 6a, ZnO, the ZnO_0.7_/TiC_0.3_ and ZnO_0.6_/TiC_0.4_ composites exhibited type IV N_2_ adsorption–desorption isotherms. The surface areas of the ZnO, ZnO_0.7_/TiC_0.3_ and ZnO_0.6_/TiC_0.4_ composites were measured to be 45, 93.5 and 90.2 m^2^/g, respectively. Due to the high surface area of Ti_3_C_2_ as reported in the literature, the surface of the ZnO composites is enhanced after integration with Ti_3_C_2_ [39]. The high surface area of the ZnO_0.7_/TiC_0.3_ composite contained abundant active sites, which consequently accelerates the surface redox reactions to produce H_2_. Interestingly, the surface area of the ZnO_0.6_/TiC_0.4_ composite with a larger content of Ti_3_C_2_ is lower in contrast to the ZnO_0.7_/TiC_0.3_ composite, which is in line with the UV-Vis and PL results and also suggests that the optimization of the Ti_3_C_2_ content is mandatory to achieve the highest surface area with the maximum number of active sites to accelerate the surface reactions.

Moreover, the band structures and charge transfer directions of the as-prepared ZnO and ZnO_0.7_/TiC_0.3_ samples were evaluated by Mott–Schottky (MS) analysis as shown in Figure 6b. The positive slopes of the MS analysis identified both candidates as n-type semiconductors with flat band potentials (E_FB_) of −1.07 and −0.74 V vs. Ag/AgCl for bare ZnO and ZnO_0.7_/TiC_0.3_ composites, respectively [36,37,38,39,40]. These values were correspondent to −0.87 and −0.54 V vs. NHE using the transformation formula: E_NHE_ = E_Ag/AgCl_ + 0.197) [38]. Therefore, owing to the equivalency of the Fermi level (E_f_) with E_FB_, the E_f_ of ZnO and ZnO_0.7_/TiC_0.3_ were found to be −0.87 and −0.54 V vs. NHE, respectively. Moreover, the value of E_FB_ is always 0.1 V higher than the conduction band potential (E_CB_) of the corresponding n-type candidate as per the literature [41,42]. Hence, the E_CB_s of the bare ZnO and the ZnO_0.7_/TiC_0.3_ composite were measured to be −0.97 and −0.64 V, respectively. It is obvious that the E_CB_ of the ZnO_0.7_/TiC_0.3_ composite is lower in contrast to bare ZnO, revealing that electrons were transmitted from ZnO to Ti_3_C_2_. By using the formula E_VB_ = E_CB_ [39], the valence band potentials (E_VB_) of ZnO and the ZnO_0.7_/TiC_0.3_ composite were determined to be 2.34 and 2.28 V, respectively, which clearly demonstrates that the E_VB_ of the ZnO_0.7_/TiC_0.3_ composite was lower in contrast to bare ZnO. The Tang group reported that the value of E_f_ for bare Ti_3_C_2_ was found to be −0.45 V vs. NHE; therefore, the integration of ZnO (E_f_ = −0.87 V vs. NHE) with Ti_3_C_2_ (E_f_ = −0.45 V vs. NHE) drives electrons from ZnO to Ti_3_C_2_ until the stability of two E_f_ is obtained with the subsequent formation of the interfacial Helmholtz double layer (HDL) between ZnO and Ti_3_C_2_ [40]. Under the circumstances of the charge redistribution process stemming from the electrostatic induction, the electrons and positive ions accumulate on the side of Ti_3_C_2_ and ZnO, respectively, to form a region devoid of free carriers, and thereby induce an electric field at the interface that cannot perturb the entire semiconductor (ZnO) because of the relatively small free carrier density in ZnO, which consequently provides a smaller number of free carriers in the domain near to the charge devoid region compared to the bulk phase of ZnO, where the charge devoid region is formed. The built-in electric field controls the transit of the charge carriers in the charge devoid region and causes band bending for the CB and VB of ZnO to prevent the backward flow of electrons, and ultimately generates the Schottky junction between ZnO and Ti_3_C_2_ [41].

**Figure 7:**
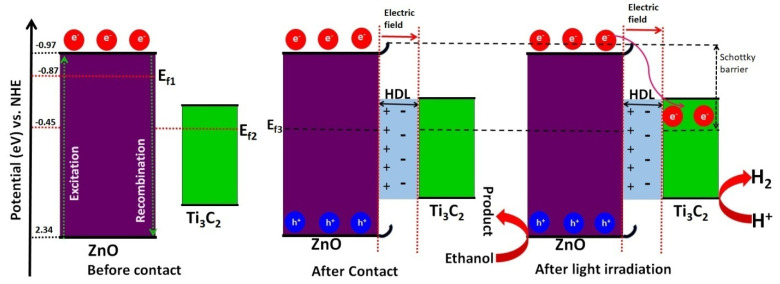
The mechanism of photocatalytic H_2_ evolution activity over ZnO_0/7_/TiC_0/3_ composite.

Based on the above discussion and the corresponding band structure of 0D ZnO and 2D Ti_3_C_2_, the plausible mechanism for the charge transfer over the 0D/2D ZnO/Ti_3_C_2_ composite for photocatalytic H_2_ evolution from a water–ethanol mixture is proposed in Figure 7. Under visible light illumination, the electrons in the valence band (VB) of ZnO after absorbing light photons with energy ≥ Eg are excited and migrate to the conduction band from the valance band of ZnO. The holes stay in the valance band of ZnO [42].

The formation of the Schottky junction traps the electrons in the CB of ZnO due to its electron sink role, hinders the counter flow of the electrons to the CB of ZnO owing to the effective influence of the built-in electric field, and thereby strongly induces the separation of excitons in the ZnO/Ti_3_C_2_ composite [43,44]. Due to the light excitation, the electrons are transferred to Ti_3_C_2_ and, due to the built-in electric field at the interface, by the Schottky junction. The electrons are then shifted to the surface of Ti_3_C_2_ due to its high electrical conductivity. Eventually, the efficient H_2_ evolution performance of Ti_3_C_2_ and the electrons built up on Ti_3_C_2_ improve the reduction of H^+^ into H_2_ [45]. Meanwhile, holes in the VB of ZnO oxidize the C_2_H_5_OH. Therefore, the oxidation of the holes via the sacrificial agent can provide numerous surface catalytic sites for photoinduced electrons migration to yield H_2_ [45,46]. In summary, the efficiently increased photocatalytic efficiency towards H_2_ by the formation of the Schottky junction can originate from the below-mentioned reasons: (i) the development of the Schottky junction between ZnO and Ti_3_C_2_ can not only cause a wide optical response towards the visible region but can also improve the separation of the charge carriers and their transmission to the surface; (ii) the intimate coupling brings a high surface area with numerous active sites; (iii) the built-in electric field effectively prevents the recombination and accelerates the transport of the charge carriers to delay their recombination rate [46,47].

## 4. Conclusions

In this study, the 0D/2D heterojunctions of ZnO/Ti_3_C_2_ were successfully synthesized using a facile electrostatic self-assembly method. The produced heterojunctions resulted in high photocatalytic hydrogen evolution in an aqueous ethanol solution under visible light exposure. The hydrogen evolution activity greatly relied on the amount of Ti_3_C_2_ added in the heterojunction system. The ZnO/Ti_3_C_2_ composite with 30 wt% loading of Ti_3_C_2_ exhibited eight times (3146 vs. 386 µmol/h/g) higher hydrogen evolution activity than the pristine ZnO. This heterojunction also showed strong stability over the pristine ZnO. The highly promoted photocatalytic efficiency was assigned to a built-in electric field due to the construction of the Schottky junction between ZnO and Ti_3_C_2_, which effectively transported the photoinduced electrons from ZnO to Ti_3_C_2_ to take part in the redox reaction. Our results revealed that the formation of the 0D/2D ZnO/Ti_3_C_2_ heterojunction system provided the spatial separation of charges and inhibited their recombination rate, proclaiming a greatly improved efficiency and stability. The present study may provide new approaches for the construction of 2D MXene and 2D semiconductor-based heterojunctions for photocatalytic and environment treatment applications.

## Data Availability

The published data is available from the corresponding author on a reasonable request.
